# Management of dysphagia and gastroparesis in Parkinson’s disease in real-world clinical practice – Balancing pharmacological and non-pharmacological approaches

**DOI:** 10.3389/fnagi.2022.979826

**Published:** 2022-08-11

**Authors:** Roongroj Bhidayasiri, Warongporn Phuenpathom, Ai Huey Tan, Valentina Leta, Saisamorn Phumphid, K. Ray Chaudhuri, Pramod Kumar Pal

**Affiliations:** ^1^Chulalongkorn Centre of Excellence for Parkinson’s Disease and Related Disorders, Department of Medicine, Faculty of Medicine, Chulalongkorn University, King Chulalongkorn Memorial Hospital, Thai Red Cross Society, Bangkok, Thailand; ^2^Academy of Science, Royal Society of Thailand, Bangkok, Thailand; ^3^Division of Neurology, Department of Medicine, Faculty of Medicine, Universiti Malaya, Kuala Lumpur, Malaysia; ^4^Department of Basic and Clinical Neurosciences, The Maurice Wohl Clinical Neuroscience Institute, Institute of Psychiatry, Psychology & Neuroscience, Parkinson’s Foundation Centre of Excellence, King’s College London, London, United Kingdom; ^5^National Institute of Mental Health and Neurosciences, Bengaluru, India

**Keywords:** Parkinson’s disease, dysphagia, gastroparesis, diagnosis, treatment, multidisciplinary team, pharmacological treatment, non-pharmacological treatment

## Abstract

Gastrointestinal (GI) issues are commonly experienced by patients with Parkinson’s disease (PD). Those that affect the lower GI tract, such as constipation, are the most frequently reported GI problems among patients with PD. Upper GI issues, such as swallowing dysfunction (dysphagia) and delayed gastric emptying (gastroparesis), are also common in PD but are less well recognized by both patients and clinicians and, therefore, often overlooked. These GI issues may also be perceived by the healthcare team as less of a priority than management of PD motor symptoms. However, if left untreated, both dysphagia and gastroparesis can have a significant impact on the quality of life of patients with PD and on the effectiveness on oral PD medications, with negative consequences for motor control. Holistic management of PD should therefore include timely and effective management of upper GI issues by utilizing both non-pharmacological and pharmacological approaches. This dual approach is key as many pharmacological strategies have limited efficacy in this setting, so non-pharmacological approaches are often the best option. Although a multidisciplinary approach to the management of GI issues in PD is ideal, resource constraints may mean this is not always feasible. In ‘real-world’ practice, neurologists and PD care teams often need to make initial assessments and treatment or referral recommendations for their patients with PD who are experiencing these problems. To provide guidance in these cases, this article reviews the published evidence for diagnostic and therapeutic management of dysphagia and gastroparesis, including recommendations for timely and appropriate referral to GI specialists when needed and guidance on the development of an effective management plan.

## Introduction

*A 68-year-old woman with a 5-year history of Parkinson’s disease (PD) had reported a slow decline in her neurological symptoms to her doctor. She described more noticeable tremor and slow gait although she was able to do most daily activities unassisted without any fall episodes. However, what she was most concerned about was not her tremor or fear of falling but, instead, the fear of eating as she felt that it was getting more difficult for her to chew and swallow her favorite Thai dish, Pad Thai – the noodles were too small for her to chew properly but too large to swallow all of them completely. When she felt the noodles were stuck in her throat, she would attempt to alleviate this sensation by rapidly drinking a big glass of water, but this would frequently lead to choking episodes. Even after only eating half her plate of food, she would feel bloated and over-full, causing her discomfort when she took a full deep breath. When she lay down, she regurgitated the food she had just eaten. Adjustment of her regular levodopa medication by increasing her daily doses reduced her tremor successfully but did not alleviate her eating concerns.*

This clinical vignette reflects the intrinsic link between the brain and gastrointestinal (GI) system, which has been acknowledged for several decades (Edwards et al., 1992; Camilleri, 2021). The bi-directional interplay of neuronal, immunological and metabolic signaling between the GI tract and central nervous system (CNS) – frequently termed the ‘gut-brain axis’ – and the influence each has on the health and pathology of the other has been the subject of considerable research in neurological diseases, including PD (Carabotti et al., 2015; Santos et al., 2019; Cryan et al., 2020). From pathological studies of alpha-synuclein deposition in the enteric nervous system to pre-clinical and clinical studies interrogating the role of the vagus nerve and gut microbiota in PD, there is growing evidence that the gut may play a fundamental role in PD aetiology and progression (Breen et al., 2019; Toh et al., 2021). Meanwhile, in the clinical setting, GI dysfunction has been recognized as having important clinical implications for patients with PD since the very first description of the disease in 1817 (Pfeiffer, 2018).

GI issues are among the most common non-motor symptoms in PD and can occur at any stage of the disease, from prodromal to advanced stages (Metta et al., 2022). They can involve different parts of the GI tract and correlate strongly with health-related quality of life (Gallagher et al., 2010; Pfeiffer, 2016). Approximately 30% of patients with PD are reported to have some kind of GI disorder (Pfeiffer, 2011), and these have been found to occur more commonly than in the general population (Chaudhuri et al., 2006; Su et al., 2017a). Despite the increase in awareness of GI issues in PD in recent years, they are still under-recognized and not considered as a priority in overall PD management.

Constipation is probably the best-known lower GI symptom, and it can occur many years before a diagnosis of PD is confirmed (Fasano et al., 2015; Schrag et al., 2015; Stocchi and Torti, 2017). Bowel-related symptoms are some of the most frequently reported and troublesome aspects of PD for patients (Politis et al., 2010). GI symptoms affecting the upper GI tract, such as swallowing dysfunction (dysphagia) and delayed gastric emptying (gastroparesis), are also common in PD; however, they are less well recognized and are, therefore, more likely to be overlooked. Notably, these symptoms can occur even in the early stages of PD (Sung et al., 2014) and become more pronounced with advancing disease, predisposing patients to increased risk of unintentional weight loss, malnutrition, frailty and mortality (Wills et al., 2016; Paul et al., 2019). Upper GI infection and dysbiosis, including small intestinal bacterial overgrowth (SIBO) and *Helicobacter pylori* infection, have also been reported to be prevalent in patients with PD and appear to be associated with worse motor function and worse PD severity (Tan et al., 2014, 2015; Leta et al., 2021). Importantly, upper GI dysfunction has been shown to affect PD medication intake and absorption, with negative implications for PD motor control (Chaudhuri et al., 2016; Pfeiffer et al., 2020).

With the close gut-brain connection in mind, the holistic management of PD should ideally include prompt and comprehensive management of GI issues. From the standpoint of ‘real-world’ clinical practice, however, PD is a complex, heterogenous and progressive condition. Management of the motor aspects alone can be challenging, often requiring multiple medications alongside other treatment strategies (Bloem et al., 2021). In the case of patients with PD who have multiple co-morbidities and treatment considerations, GI issues may easily go unnoticed or may be perceived as less of a priority; some clinicians may consider managing GI issues only after motor problems have been resolved. This is a fundamental misconception as GI and motor issues in PD are intrinsically linked and need to be dealt with together. Furthermore, treatment of PD motor symptoms with dopaminergic drugs and other medications (e.g., those with anticholinergic effects) can also cause or exacerbate some of these GI issues.

While management of these GI issues generally falls outside the usual remit of neurologists, it is important that they are aware of them and their prevalence in patients with PD and how they could potentially be treated. Developing an increased awareness of these GI problems is likely to increase timely referral to appropriate specialists for early intervention and ultimately better quality of life for patients. Neurologists can also provide early information on possible treatment approaches, which can then be implemented by GI specialists, PD nurse specialists and speech and swallowing therapists. If left untreated, not only are these GI issues distressing for patients, but they can also impact the delivery to and absorption of oral levodopa and other medications from the small intestine, which ultimately can have a negative effect on motor symptom control, and also lead to life-threatening complications, such as aspiration pneumonia or intestinal perforation (Nyholm and Lennernas, 2008; Pfeiffer et al., 2020).

Although a multidisciplinary and collaborative approach to the management of GI issues in PD is recommended, this is not always possible in every setting, often resulting in low referral rates (Shrubsole, 2021). In ‘real-world’ practice, neurologists are often the ones at the forefront with patients who are experiencing these types of problems (Bhidayasiri et al., 2020). There is increasing evidence that PD nurse specialists can play a pivotal role as part of the wider PD care team by supporting patients and their carers, assessing and monitoring symptoms, and coordinating care with the neurologist and other specialists (Tenison et al., 2022). This article focuses on two of the common, but often overlooked, upper GI clinical features that occur in patients with PD: dysphagia and gastroparesis. It provides an overview of the published evidence regarding their diagnostic and therapeutic management, including recommendations for timely and appropriate referral to GI specialists when needed and guidance on the development of an effective management plan considering both pharmacological and non-pharmacological options. This balance of pharmacological and non-pharmacological approaches is key, as in many cases, patients with PD find pharmacological approaches alone for these types of GI issues are either only partially effective or totally unsatisfactory. Non-pharmacological approaches are, therefore, often the cornerstone and first-line treatment for the management of dysphagia and gastroparesis in PD. To provide optimal management of GI issues in PD, both pharmacological and non-pharmacological approaches need to be implemented together.

## Dysphagia in Parkinson’s disease

In healthy individuals, the act of swallowing occurs in several defined phases (Table 1), each of which involves multiple muscles along with complex neural control and coordination: the oral phase (chewing and propulsion of food to the pharynx), the pharyngeal phase (moving the bolus of food through the pharynx to the esophagus, including the closure of the airway), and the oesophagal phase (transport of the food from the esophagus into the stomach) (Malandraki and Robbins, 2013; Patel et al., 2020).

**TABLE 1:** Physiology of normal swallowing in healthy individuals and pathophysiology of dysphagia in patients with Parkinson’s disease (PD) according to phases of swallowing.

Phases of swallowing	Normal swallowing in healthy individuals	Swallowing abnormalities in patients with PD who have dysphagia
Oral phase	Chewing and propulsion of food to the to the pharynx.	• Impaired chewing. • Impaired jaw opening. • Impaired oral stereognosis.
Pharyngeal phase	Moving the bolus of food through the pharynx to the esophagus, including closure of the airway.	• Delayed swallowing reflex. • Prolongation of the pharyngeal transit time. • Repetitive swallows to clear the throat.
Esophageal phase	Transport of the food from the esophagus into the stomach.	• Impaired motility of the smooth muscle esophagus. • Impairment of the esophageal bolus and hypo- or aperistalsis in early stage of PD, increased peristalsis in advanced stage.

Dysphagia describes the deficits that can occur in the normal functioning of any of these three phases. These deficits can impact not only swallowing efficiency and delivery of food to the stomach but also swallowing safety. If the airway is not closed promptly and efficiently, there is a risk of food aspiration, which can lead to aspiration pneumonia. Dysphagia severity has also been identified as a predictive factor for poor outcomes in patients with late-stage PD (Fabbri et al., 2019). A recent review has highlighted the considerable direct and indirect impact that PD can have on oral health and hygiene and the importance of considering oral health as a part of PD management since it is a critical component of overall health, well-being and quality of life for patients (Auffret et al., 2021). Oral health has also been shown to be a predictive factor for the risk of aspiration pneumonia in PD (Noguchi et al., 2018).

### Prevalence

The reported prevalence of dysphagia in the PD population is likely to be underestimated as, particularly in early-stage PD, it can be relatively asymptomatic (Sung et al., 2010). Although the overall prevalence of dysphagia increases with age, it has been found to be more prevalent in people with PD than in the general population (Edwards et al., 1992). Reports suggest that >80% of patients with PD develop dysphagia during the course of their disease and that it can occur at any stage of PD, not just when the condition becomes advanced, including at the prodromal stage and before the classic motor PD symptoms become apparent (Suttrup and Warnecke, 2016). However, patients with PD often do not report or do not perceive that they have, swallowing difficulties when in fact, subsequent objective clinical tests confirm they have some degree of dysphagia (Kalf et al., 2012; Pflug et al., 2018).

In the initial oral phase of swallowing, chewing issues are common in patients with PD but, in many cases, are overlooked as the focus is often on the act of swallowing itself. As chewing occurs before swallowing, any deficits here will make it more difficult for patients to swallow. Studies have shown that patients with PD report more problems with their oral health, including issues with chewing and swallowing than the general population, with increased prevalence seen in those with longer and more severe disease; about one-third of patients with PD were reported to have issues with chewing and biting in one study (Nakayama et al., 2004; van Stiphout et al., 2018). Male gender and cognitive impairment were recently identified as predictors of dysphagia in PD (Wang et al., 2021). Indeed, the contribution of cognition to effective swallowing was evident when swallowing was found to be significantly impaired under attention-demanding cognitive and motor dual-task interference (Ardenghi et al., 2021; Labeit et al., 2021). These findings highlight the fact that swallowing function is not purely reflexive but requires mental capacity.

A meta-analysis of the prevalence of oropharyngeal dysphagia in patients with PD illustrates a disconnect between patient-reported (subjective) and clinician-assessed (objective) outcomes. Analysis of data from 10 studies that used subjective outcome measures found a pooled prevalence estimate of 35%, while four studies that used objective outcome measures found a pooled prevalence estimate of 82%; prevalence in control subjects was found to be 9% (Kalf et al., 2012). A study of pharyngoesophageal activity in 54 patients with early-stage PD (<3 years from diagnosis) using oesophagal manometry in the liquid swallow and viscous swallow tests found that the majority of patients showed pharyngeal and oesophagal dysfunction even before clinical manifestations of dysphagia (Sung et al., 2010).

Dysphagia has also been reported in various atypical parkinsonian disorders (APDs), including multiple system atrophy (MSA) (Higo et al., 2003; Taniguchi et al., 2015; Lee et al., 2018; Warnecke et al., 2019), progressive supranuclear palsy (PSP) (Warnecke et al., 2010) and Lewy body dementia (LBD) (Hely et al., 1996; Muller et al., 2001). In fact, dysphagia within 1 year of disease onset is recognized as a distinguishing feature of APDs versus PD in which it generally has a longer latency time (Hely et al., 1996; Muller et al., 2001; Suttrup and Warnecke, 2016; Bhidayasiri et al., 2019; Umemoto and Furuya, 2020).

### Pathophysiology

**FIGURE 1:**
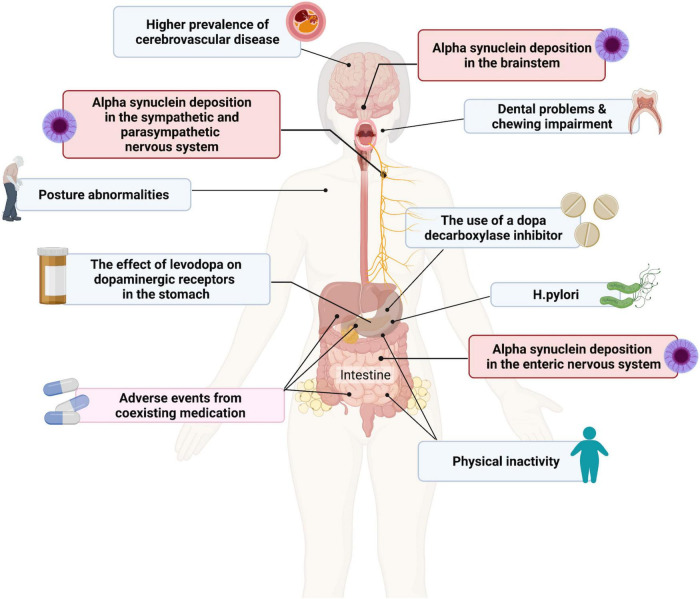
Contributing factors to dysphagia and gastroparesis in patients with Parkinson’s disease (PD). Created with BioRender.com.

The underlying pathophysiology of dysphagia in PD remains to be fully elucidated and is likely to be multifactorial in origin and involve both dopaminergic and non-dopaminergic mechanisms (Figure 1) (Malandraki and Robbins, 2013; Suttrup and Warnecke, 2016; Patel et al., 2020). It has been suggested that dysphagia may be a secondary effect of progressive neurodegeneration in PD on the oropharyngeal muscles or dysfunction of the normal coordination of the oropharyngeal and oesophagal muscles due to brainstem involvement (Sung et al., 2010). It can also be a clinical manifestation of motor fluctuations. Alpha-synuclein deposition in the ENS and in nerves innervating the pharyngeal muscles have also been reported, but their contribution to dysphagia is as yet unknown (Suttrup et al., 2017; Patel et al., 2020).

Both PD and cerebrovascular disease (CVD) are more common in older age, but studies have suggested a direct association between these two conditions (Winikates and Jankovic, 1999). A post-mortem study of consecutive cases of autopsy-proven idiopathic PD (*n* = 617) and age-matched controls (*n* = 535) found that the frequency of cerebrovascular lesions (including amyloid angiopathy, white matter lesions, ischemic infarcts and hemorrhages) was higher (44.0%) in subjects with PD than in controls (32.8%) (Jellinger, 2003). A meta-analysis of stroke risk in patients with PD showed a close correlation between the two disorders and possibly common pathogenesis (Liu et al., 2020). A similar higher prevalence of radiologically- and clinically-confirmed CVD has been observed in patients with PD compared to controls and this has the potential to impact the clinical presentation and prognosis of PD, which could include an increased risk of dysphagia (Nanhoe-Mahabier et al., 2009; Patel et al., 2011). However, a direct association between CVD in PD and dysphagia has not been established.

### Impact of dysphagia on patients with Parkinson’s disease

Untreated dysphagia can have significant negative consequences for people with PD (Malandraki and Robbins, 2013; Suttrup and Warnecke, 2016). From a clinical standpoint, probably the most serious of these is the risk of food aspiration with subsequent development of pneumonia, which is known to be the leading cause of death in patients with PD (Dilmaghani et al., 2022). However, there are also implications for the patient’s overall quality of life, and the possible stigma or embarrassment of coping with swallowing problems, which are often accompanied by drooling, in social situations (van Wamelen et al., 2020). Over the longer term, a reduced ability to swallow normally may result in malnutrition, dehydration, and unintentional weight loss (van Wamelen et al., 2020).

Importantly for patients with PD, dysphagia can impact the ingestion and clinical efficacy of levodopa and other oral PD medications. A case report illustrated the retention of oral levodopa tablets in the epiglottic vallecula in a patient with PD, and how effective swallowing strategies can help improve symptoms of motor fluctuations (Sato et al., 2018). A retrospective analysis of endoscopic data for swallowing function in patients with PD found that those who experienced a delayed onset of clinical effect (delayed ON) after taking a dose of oral medication more commonly had pooling of saliva, a delayed swallowing reflex, and residual drug in the pharynx than those who did not experience delayed ON (Fukae et al., 2020). Therefore, proactive assessment and management of dysphagia in patients with PD are needed due to its potential serious clinical consequences and overall negative impact on QoL, medication efficacy, and nutritional status. The most appropriate intervention will also depend on the particular nature of the dysphagia impairment, namely whether it is primarily oral, pharyngeal, or oesophagal.

### Recognition and assessment

A standardized phenotypic classification system for neurogenic dysphagia has been developed, recognizing that dysphagia is not a discrete symptom, but a multifactorial syndrome with different phenotypic patterns that can emerge depending on the underlying disease (Warnecke et al., 2021). Common characteristics of dysphagia in PD include:

• Chewing problems, poor dental health.
• Pharyngeal residue, sometimes reported in nearly all patients, often reported as the lead finding, with predominance in the valleculae.
• Premature bolus spillage.
• Impaired swallowing reflex.
• Pharyngolaryngeal movement disorders.
• Impaired secretion management (rarely reported).

Various techniques have been proposed for the assessment and diagnosis of dysphagia in PD as well as in other neurological conditions (Nishiwaki et al., 2005; Lam et al., 2007; Wieseke et al., 2008; Bours et al., 2009; Suttrup and Warnecke, 2016; Patel et al., 2020). However, many of these techniques are quite specialized and may be unfamiliar to neurologists in their daily practice, especially those not in major healthcare facilities or teaching hospitals. Here, we highlight some of the simpler, minimally invasive techniques that can be undertaken at the patient’s bedside or in the clinic that can provide useful initial information to help identify patients who may need additional investigations and guide appropriate referrals for the more specialized techniques. The simplest and most commonly used initial screening assessments are patient questionnaires and bedside swallowing evaluations. They can often be undertaken by appropriately trained nurses and can be used to help identify patients who may warrant further, more invasive instrumental testing (Suttrup and Warnecke, 2016).

Validated and PD-specific swallowing questionnaires are available, including the Swallowing Disturbance Questionnaire (SDQ) and Munich Dysphagia test–Parkinson’s disease (MDTPD) (Table 2) (Patel et al., 2020). Due to increased recognition of dysphagia even in early PD, the MDTPD questionnaire has been specifically validated for this purpose (Simons et al., 2014). Recently, the Gastrointestinal Dysfunction Scale for PD (GIDS-PD) has been developed as a self-report tool to quantitatively assess the presence and severity of GI dysfunction features, including dysphagia and sialorrhea, in patients with PD with strong reliability and validity (Camacho et al., 2021). Generic non-motor questionnaires, including Non-Motor Symptoms Questionnaire (NMSQuest) and Non-Motor Symptoms Scale (NMSS) may be helpful in this setting as it enables healthcare teams to determine the presence a diverse range of NMS, including GI issues, so that they can be referred for further investigation or treatment (Chaudhuri et al., 2006, 2007).

**TABLE 2:** Validated questionnaires for upper GI tract evaluation.

Scales	Components
Swallowing Disturbance Questionnaire (SDQ) Likert scales rated by patients 0: no disturbance 1: mild disturbance 2: moderate disturbance 3: severe disturbance except for the last one: Yes was scored 2.5 and no was scored 0.5)	- Fifteen questions (1) Do you experience difficulty chewing solid food, like an apple, cookie or a cracker? (2) Are there any food residues in your mouth, cheeks, under your tongue or stuck to your palate after swallowing? (3) Does food or liquid come out of your nose when you eat or drink? (4) Does chewed-up food dribble from your mouth? (5) Do you feel you have too much saliva in your mouth; do you drool or have difficulty swallowing your saliva? (6) Do you need to swallow chewed-up food several times before it goes down your throat? (7) Do you experience difficulty in swallowing solid food (i.e., do apples or crackers get stuck in your throat)? (8) Do you experience difficulty in swallowing pureed food? (9) While eating, do you feel as if a lump of food is stuck in your throat? (10) Do you cough while swallowing liquids? (11) Do you cough while swallowing solid foods? (12) Do you experience a change in your voice, such as hoarseness or reduced intensity immediately after eating or drinking,? (13) Other than during meals, do you experience coughing or difficulty breathing as a result of saliva entering your windpipe? (14) Do you experience difficulty in breathing during meals? (15) Have you suffered from a respiratory infection (pneumonia and bronchitis) during the past year?
Munich Dysphagia test-Parkinson’s disease (MDTPD) Likert scales rated by patients 0: never, 1: occasionally, 2: often, 3: very often except for the last sub-scale; Yes was scored 0 and no was scored 3.)	- Twenty-six questions with four sub-scales: (1) Difficulty swallowing food and liquids (10 items): items are organized by the sequence of the oropharyngo-esophageal swallowing act and potential pathologies. (2) Difficulty swallowing independent from food intake (4 items): includes sialorrhea (drooling), (drug-related) xero- stomia, saliva penetration/aspiration, and difficulties during pill intake, e.g., aggravated oropharyngeal transport. (3) Further swallowing-specific and accompanying burden (nine items): negatively influences the swallowing-related daily routine, and amplifies already acquired symptoms. Focuses are set on motor fluctuations and patients compensation behavior to swallow easier or avoid the intake of specific consistencies. (4) Swallowing-specific health questions (three items): for acquiring medically relevant information on dysphagia or health risks.
Gastroparesis Cardinal Symptom Index (GCSI) Likert scales rated by patients 0: none, 1: very mild, 2: mild, 3: moderate, 4: severe, 5: very severe)	- Nine questions. - Please rate the severity of the following symptoms during the past 2 weeks. (1) Nausea. (2) Retching. (3) Vomiting. (4) Stomach fullness. (5) Early satiety. (6) Postprandial fullness. (7) Loss of appetite. (8) Bloating. (9) Stomach distension.

**FIGURE 2:**
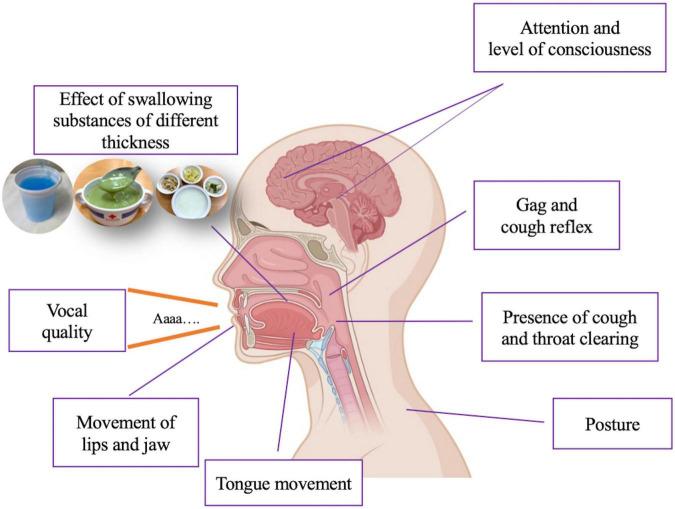
Assessment of dysphagia in clinical practice. Created with BioRender.com.

A clinical swallowing evaluation can be undertaken at the bedside and generally includes asking questions about the nature of any swallowing problems, a physical examination of the head and mouth and a practical assessment of oral function. Ideally, this assessment should be performed by speech therapists; however, in real-world clinical practice, this evaluation can be performed quite safely by either a neurologist or PD nurse specialist at the patient’s bedside. This practical test can comprise an assessment of movement (lips and jaw), reflexes (gagging and coughing), vocal quality, presence of cough, and throat clearing, and the effect of swallowing substances of different thickness (water, pureed food, and solid food) (Figure 2). The swallowing test focuses on spillage of substances from the mouth, residue or pocketing of material in the mouth, and chewing efficiency. The patient’s level of oxygen desaturation can also be a useful parameter to evaluate aspiration or penetration of substances into the airway. A systematic review undertaken in 2009 to determine the effectiveness and feasibility of bedside screening methods for detecting dysphagia in patients with neurological disorders concluded that the water swallowing test combined with pulse oximetry using coughing, choking and voice alteration as the endpoints was the best strategy in that setting (Bours et al., 2009). For example, the patient may be asked to swallow 50 ml of water in 10 ml aliquots with the tester assessing coughing or choking, a change in voice quality or a change in oxygen desaturation of >2%.

While such bedside tests are not always standardized and validated and should not be used as the sole means of diagnosing dysphagia, they can be extremely useful as an initial step in the identification of dysphagia and can be relatively easily undertaken in clinical practice. More detailed investigations using instrumental techniques are likely to provide more reliable results but require to be undertaken by trained personnel, including, amongst others, speech and language therapists, radiologists, and ENT specialists (Patel et al., 2020). These techniques include:

• Video fluoroscopic swallow study (VFSS)/modified barium swallow study (MBSS).
• Fiber-optic endoscopic evaluation of swallowing (FEES).
• High-resolution manometry (HRM).

### Developing a management plan

**FIGURE 3:**
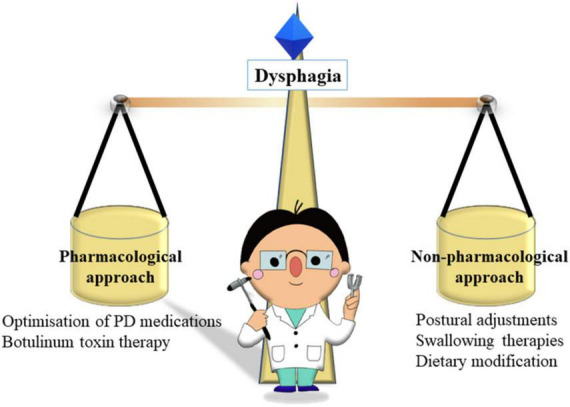
The roles of neurologist in balancing pharmacological and non-pharmacological approaches in dysphagia.

The management of dysphagia in patients with PD should ideally take a holistic and multidisciplinary approach, considering strategies to improve the physical/mechanical aspects of swallowing, including compensatory strategies when eating and drinking, modifications of diet, physical exercises to strengthen musculature, as well as medication options (Figure 3) (Lopez-Liria et al., 2020; Patel et al., 2020; Umemoto and Furuya, 2020; Schindler et al., 2021). However, there is much that individual neurologists can do to initiate the management plan and to help patients achieve adequate, safe swallowing and to minimize the risk of aspiration.

Many of the strategies suggested for the rehabilitation of dysphagia in patients with PD have, in fact, been developed from those used successfully in stroke patients, but not all are suitable for a progressive disease like PD (Umemoto and Furuya, 2020). An international group of experts in the field of neurogenic dysphagia and PD has recently conducted a systematic literature review and developed a set of consensus statements on the treatment of dysphagia in PD (Schindler et al., 2021). They concluded that indications for dysphagia treatment are as follows:

• Treatment should be started when there is clinical or instrumental evidence of impairment of swallowing safety and/or swallowing efficiency and/or reduced QoL, irrespective of the stage of the disease.
• When patients with PD require treatment for dysphagia, an instrumental assessment of swallowing is indicated to guide the treatment plan.

The consensus was that treatment options could include optimization of antiparkinsonian treatments, dietary modifications and fluid thickening, strategies for nutritional management, postures, swallowing maneuvers, swallowing exercises, neuromodulation, and medical treatments. As mentioned, dysphagia can be a clinical manifestation of motor fluctuations, so if dysphagia is observed to fluctuate with motor symptoms, then optimization of dopaminergic therapy is essential. Some of these strategies can be initiated by the treating neurologists, while others will require specialist intervention. Summarized here are some initial strategies that have been investigated and may be considered by clinicians or recommended to patients and carers, along with information about their relative efficacy based on published literature.

#### Compensatory management

Compensatory strategies focus on the implementation of relatively simple techniques to facilitate the safe oral intake of foods or liquids or to provide alternate sources of nutrition to ensure the maintenance of good nutritional status. Such strategies include educating the patient about their condition, postural adjustments when eating, different eating techniques and swallowing maneuvers, and modifications to the diet.

##### Postural adjustments

Adjustment of the patient’s head or body positioning while eating has been suggested to help protect the airway and reduce aspiration of food; however, there is some debate as to their benefits over the longer term and their overall value in preventing nutritional deficits (Patel et al., 2020; Schindler et al., 2021).

Patients with PD often have a stooped posture and also a tendency to lean to one side, especially during OFF periods that can occur between medication doses. In addition, reduced spatial awareness may partly account for poor swallowing amongst patients with PD. The Alexander Technique may help improve day-to-day movement in PD, and there is evidence that instruction in Alexander Technique can lead to sustained benefits in patients with PD (Stallibrass et al., 2002). Other methods for addressing poor posture include encouraging improved awareness of poor posture with verbal prompts to ‘straighten up’ or the use of a high-backed chair to give a physical prompt to the head; chair raisers and powered riser–recliner type chairs may suit some patients. If dyskinesia occurs when sitting in an armchair before eating, there may be a risk of sliding forwards and possibly falling out of the seat. For mild-to-moderate dyskinesia, a one-way glide sheet or latex netting placed on the seat cushion may be enough to give additional resistance. For more significant dyskinesia, a deep pressure-relief foam cushion with a ramped front edge, secured with non-slip material, may be useful when placed on the chair cushion (Aragon and Kings, 2010).

**FIGURE 4:**
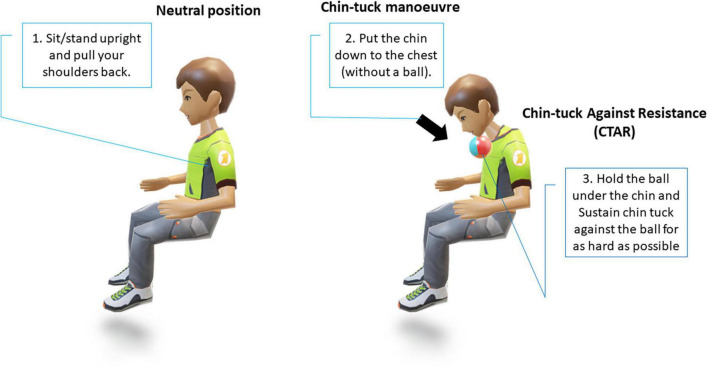
Procedure of chin-tuck maneuver and chin-tuck against resistance.

The chin-tuck maneuver, also known as the chin-down posture (Figure 4), is a commonly used compensatory strategy; however, its reported effectiveness in studies in patients with PD with dysphagia has varied (Ra et al., 2014; Saconato et al., 2016). These early studies often lacked clear objective markers, however, more recent evidence from studies using objective outcomes has demonstrated that it has a significant benefit in reducing laryngeal penetration in patients with PD with dysphagia (Ko et al., 2021). However, in real-world clinical practice, while the chin-tuck maneuver has proven benefits and is relatively easy to explain, for patients with PD who may have existing postural problems or tremors, it may be challenging initially (Schindler et al., 2021). Similar positive results have also been observed in stroke patients, where it has been reported that performing a chin-tuck maneuver against resistance is beneficial for improving the swallowing function of patients with post-stroke dysphagia. However, this technique requires a degree of strength and range of motion of the hand in order to hold the elastic object in place and maintain proper posture. To address this, a less physically-demanding modified chin-tuck exercise has more recently been shown to reduce aspiration and improve the nutritional status of patients with post-stroke dysphagia (Kim and Park, 2019).

##### Swallowing therapies

**FIGURE 5:**
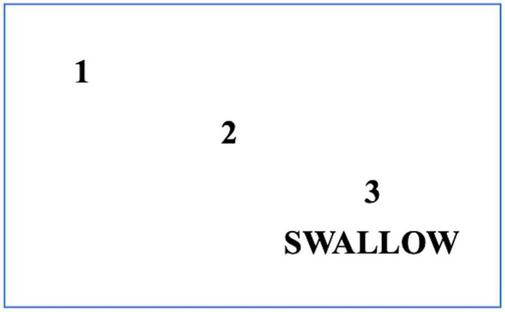
Modified eating and drinking equipments for patients with PD. **(A)** The plate mat – a non-slip material patients can put under a plate or bowl to keep it from moving on the table, **(B)** the plate guard – a 2.5 cm acrylic or metal border that fits onto a plate. Patients push the food up against the guard, and it falls onto the eating utensil, **(C)** the one-way valve straw, **(D)** the nosey cup, **(E)** the spoon–fork (Permission obtained from https://www.parkinson.org), and **(F)** the anti-choking mug with a unique design of an inner slope to allow an individual user to drink from this mug without bending the neck or tilting the head. A valve inside the mug allows a maximal amount of 10 mL per sip of water.

**FIGURE 6:**
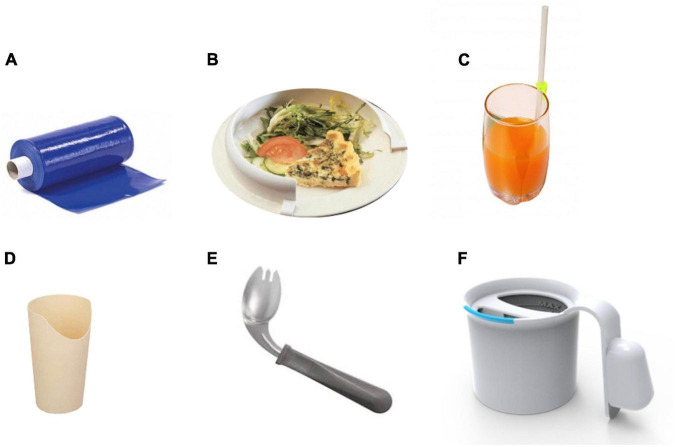
A cue card for patients with PD who have swallowing initiation problems. Patients should read this card silently and follow the numbers to the word SWALLOW. When patients reach the word ‘SWALLOW,’ they are advised to swallow food or drink simultaneously.

Referral to swallowing therapists should be considered when the patient has difficulties with swallowing and has coughing episodes when eating food or taking tablets. This situation occurs due to a reduction in the frequency with which patients with PD automatically swallow saliva as it is produced. Improving posture will help to some extent, as will developing the habit of swallowing for patients. In addition to posture, it is important to consider adequate lighting and as few distractions as possible when eating or drinking. Patients with cognitive impairment should concentrate on their swallowing and avoid performing concomitant tasks during eating (Ardenghi et al., 2021). Using a cue card may be beneficial with instructions for patients with PD to read the cue card silently and aim to commence swallowing on reading the word swallow on the card (Figure 5). Other simple maneuvers like ‘double swallow’ can be used by asking patients to perform a second swallow after their first attempt with food to ensure that all food pieces are clear from the oral cavity before a subsequent swallow with food. The patient should also be assessed if they might need modified eating and drinking equipment; for example, weighted cutlery sometimes helps dampen a tremor that persists during movement (Aragon and Kings, 2015; Bhidayasiri et al., 2022) (Figure 6). These include plate mats, bendable straws, cut-away mugs (also known as nosey cups), modified cutlery (i.e., fork knife and spoon-fork), or the innovation of an anti-choking cup with a modified angle and slope within the cup that allows an individual user to drink without bending the neck or tilting the head; thus, reduce their risk of aspiration (Figure 6).

Variations in the mechanics of swallowing in patients with PD have been found to influence overall swallowing efficacy and safety and therefore offer a possible therapeutic target for improvement. The importance of the mechanics of swallowing in dysphagia was demonstrated in a study of 40 patients with early to mid-stage PD. Swallowing kinematics were assessed using video fluoroscopic swallow studies (VFSS), and their influence on pharyngeal residue, penetration of food into the airway, and aspiration was determined (Curtis et al., 2020). The authors found that disease severity alone was not able to predict swallowing changes in PD; however, swallowing kinematics significantly influenced swallowing efficacy and safety. It was noted, however, that these kinematic variables themselves differed according to the swallowing conditions, for example, bolus size and consistency.

The use of different swallowing techniques and muscle-strengthening exercises have been employed with the aim of improving swallowing function in people with dysphagia (Schindler et al., 2021). Video-assisted swallowing therapy, whereby dysphagic patients with PD are shown a video of the usual swallowing process as well as of their own technique as part of therapy sessions, has been reported to be associated with improved swallowing-related QoL and fewer food residues in the pharynx (Manor et al., 2013). While effective, this may be difficult to implement in clinical practice.

Neuromuscular stimulation techniques have been employed in both PD and stroke patients with dysphagia and aim to improve neural functioning and muscular strength, often as an adjunct to swallowing therapies (Park et al., 2012, 2016; Schindler et al., 2021). They comprise non-invasive brain stimulation (NIBS) and transcutaneous electrical stimulation (TES), but there is limited evidence to date for their efficacy in dysphagic patients with PD. One study compared the effects of traditional dysphagia treatment with or without TES as an adjunct to therapy on the quality of life of patients with PD with dysphagia but was unable to find a significant difference between the treatment groups (Heijnen et al., 2012). However, a small, randomized, single-blind, placebo-controlled trial found that that TES applied to the infrahyoid region combined with effortful swallowing was able to increase hyoid bone movement and reduce aspiration in dysphagic patients with PD (Park et al., 2018). A meta-analysis of available evidence of the effects of TES on swallowing disorders reported that there was no firm evidence to draw conclusions regarding the efficacy of neuromuscular electrical stimulation on swallowing disorders (Sun et al., 2020). These findings are supported by the overall consensus of the international expert group that TES cannot currently be recommended for dysphagia management in PD based on the available evidence (Schindler et al., 2021).

##### Dietary modifications

Patients with PD and their carers should also be provided with advice on making dietary modifications to make mealtimes easier and eating and drinking safer. These can include:

• Carefully plan meals and snacks to ensure that they are in accordance with the recommended diet for that particular patient’s swallowing problems.
• Thicken liquids (using a commercial thickener or pureed fruit) where possible, as this will make them less likely to trickle down the throat and cause coughing, choking or aspiration. However, note that the degree of thickness required will depend on the individual patient’s ability to chew and swallow.
• Add moisture to food by using gravies or sauces.
• Ensure the patient is in a good upright position to eat their meal or snack and in their best ‘ON’ state.
• Avoid distractions and ensure there is complete focus on chewing and swallowing.
• Ensure meals are not rushed, and there is plenty of time for chewing and swallowing.
• Be patient, giving the patient as much autonomy as possible and adequate time to finish their meal.
• Prepare smaller meals or even finger foods that encourage eating but are less challenging.
• Be alert to signs of choking if food is retained in the mouth.
• Handfeeding may be needed in the case of advanced dysphagia.

#### Medical management

Medical management of patients with PD with dysphagia should include optimization of existing PD medications to ensure motor function is controlled effectively, which may entail considering alternative treatment formulations, as well as discussing other specific pharmacological therapies for dysphagia (Patel, 2015; Schindler et al., 2021).

It is suggested that patients with PD with dysphagia should be eating meals when in their best ON state, so oral PD medications should be taken approximately 30 min before mealtimes (Schindler et al., 2021). Patients may wish to consider several smaller, more frequent meals throughout the day. Where possible, oral medication regimens should be simplified to reduce the pill burden in patients with dysphagia. In terms of formulation, if oral tablets are proving difficult, dispersible options may be available, or non-oral therapies that avoid the GI route can be considered.

Data regarding whether dopaminergic PD medications can improve dysphagia are equivocal (Gandhi and Steele, 2022). It has been suggested for many years that swallowing dysfunction is not solely related to nigrostriatal dopamine deficiency and may have a non-dopaminergic influence (Hunter et al., 1997), which may explain the observed lack of response to dopaminergic medications in some cases. A systematic review of interventions for dysphagia identified nine studies that had evaluated the efficacy of different dopaminergic medications (levodopa, carbidopa, apomorphine, domperidone, and rotigotine) with mixed results (Gandhi and Steele, 2022). A study undertaken in 15 patients with advanced PD whose motor symptoms were responsive to levodopa only found clinically relevant, objective improvement of dysphagia after oral administration of levodopa and evaluation using FEES in about 50% of subjects (Warnecke et al., 2016). Similar, relatively low rates of dysphagia response to levodopa (2 out of 7 patients) have been reported using the same FEES–levodopa test in patients with PSP (Warnecke et al., 2010). Rotigotine has also been evaluated for the management of dysphagia in PD. A small, retrospective open-label study was able to show using VFSS assessment that rotigotine transdermal patch improved swallowing in six consecutive dysphagic patients with PD (Hirano et al., 2015).

Device-aided therapies, such as deep brain stimulation (DBS) and levodopa–carbidopa intestinal gel (LCIG) infusion, have also been evaluated for their effect on dysphagia in PD. Although some positive results have been reported with DBS on swallowing function in patients with PD, robust, high-quality evidence is lacking (Silbergleit et al., 2012; Chang et al., 2021). In addition, it has been suggested that any beneficial effect may wear off with long-term use (Xie et al., 2018). A retrospective study described some benefits of LCIG infusion on pharyngeal dysphagia, although this was only undertaken in a small number of patients (Labeit et al., 2020).

Botulinum toxin therapy is a new technique that is being evaluated for the management of some forms of dysphagia. The recent consensus statement on dysphagia management advises that botulinum toxin injection may be an option to treat patients who have well-documented upper esophageal sphincter (UES) impairment, in the absence of other significantly impaired swallowing mechanisms or where the UES impairment can be considered as the main pathophysiological mechanism of dysphagia based on instrumental findings (Schindler et al., 2021). It is important to note that botulinum toxin injection should only be administered by specialists who are experienced with this technique and when other simple interventions have been attempted with unsatisfactory results. Side effects, including worsening of reflux and temporary worsening of dysphagia, have been reported (Wei, 2022).

Patients with PD who experience significant dysphagia may be candidates for a non-oral therapy to manage their motor symptoms in order to completely avoid the oral route of administration. Options include a transdermal rotigotine patch, one of the newer on-demand inhaled or sublingual products recently approved in the United States, and infusion therapies or DBS. Clinical judgment is needed to determine what medications would be appropriate for each individual patient.

As aspiration pneumonia has been reported as the commonest cause of death in patients with PD, dysphagia is an important prognostic indicator, and referral to a Palliative Care specialist should be considered (Miyasaki and Kluger, 2015). Management of dysphagia in advanced patients with PD should be discussed within the context of the patient’s overall functional status and individualized according to patient’s preference and goals of care. Although the general emphasis should be in maximizing efforts to continue oral feeding, enteral feeding may be considered when prolonged and difficult mealtimes reduce the quality of life, when dysphagia has advanced out of proportion to other signs of disease progression, or if artificial nutritional support is consistent with goals of care (Lum and Kluger, 2020). However, given the lack of evidence of enteral feeding in preventing aspiration or prolonging survival, there remains significant uncertainty among neurologists regarding the appropriateness and timing of enteral feeding (Stavroulakis and McDermott, 2016; Choo et al., 2020). Notably, considerable variability in patients’ preferences has also been reported – in one Asian study, about 60% considered tube feeding versus 20% in another Caucasian study (Kwak et al., 2014; Choo et al., 2020). Taken together, a shared decision-making process involving the patient, the carers and the healthcare team is important and is required to ensure the best delivery of dysphagia care.

## Gastroparesis in Parkinson’s disease

Gastroparesis is the term used to describe delayed gastric emptying that occurs in the absence of a mechanical obstruction (Schol et al., 2021). A joint consensus statement on gastroparesis, developed using a rigorous Delphi process, has recently been published by two professional organizations in the gastroenterology field: United European Gastroenterology (UEG) and the European Society for Neurogastroenterology and Motility (ESNM), in order to develop a European consensus on the definition, clinical characteristics, pathophysiology, diagnosis, and management of gastroparesis across all patient populations (Camilleri et al., 2021; Schol et al., 2021).

Nausea and vomiting are recognized as the cardinal symptoms of idiopathic gastroparesis. In addition, dyspeptic symptoms, such as postprandial fullness, early satiation, epigastric pain, as well as bloating in the upper abdomen and belching, are often present in subjects with gastroparesis. In some cases, subjects may have delayed gastric emptying even if they are not displaying overt GI symptoms.

### Prevalence

In people with PD, gastroparesis is one of the most commonly observed GI problems, and PD is estimated to account for 7.5% of all cases of gastroparesis (Marrinan et al., 2014). The true prevalence of gastroparesis in PD is uncertain. A systematic literature review has estimated the prevalence of gastroparesis is 70–100% in patients with PD, although many cases are asymptomatic, contributing to the lack of recognition of this condition (Heetun and Quigley, 2012; Fasano et al., 2015). However, most studies reporting a high prevalence of gastroparesis in PD were based on breath test studies, and these results have not been confirmed by gastric scintigraphy studies, which are considered the gold standard for diagnosis (Knudsen et al., 2018).

Gastroparesis can occur at any stage of PD: in newly-diagnosed patients with PD as well as in those with advanced disease (Heetun and Quigley, 2012). While it is generally more prevalent in advanced stage, studies have not shown any correlation between the occurrence of gastroparesis and the duration of a person’s PD diagnosis (Heetun and Quigley, 2012). In a study of gastric emptying time in 22 patients with PD, it was found that the prevalence of delayed emptying (defined as >61 min) in patients with mild disease (48.3%) was not significantly different to that in patients with moderate disease (36.4%) (Hardoff et al., 2001).

A radionuclide gastric emptying study found that patients with PD had prolonged gastric emptying times when measured after 60 min compared with control subjects, but in addition, this delay was more marked in PD subjects who experienced motor response fluctuations (Djaldetti et al., 1996). It is likely that gastroparesis contributes to motor fluctuations in patients with PD. Evidence from a study of 31 patients with PD showed a significant correlation between levodopa pharmacokinetics and gastric emptying in patients with PD, suggesting that delayed gastric emptying is a causative factor for producing delayed-ON in PD (Doi et al., 2012). These studies highlight the importance of clinicians being vigilant for this and other GI symptoms throughout the PD patient’s journey.

### Pathophysiology and contributing factors

As for dysphagia, the underlying pathophysiology of gastroparesis in PD is not fully understood but is likely to be multifactorial (Figure 1). Other comorbid conditions, such as diabetes, may also play a role in the development of gastroparesis in PD. It may also be due in part to Lewy body pathology in the vagus nerve, which plays a central role in the neural control of gastric emptying, and in the wider ENS, as well as alpha-synuclein deposition in the brainstem (Hardoff et al., 2001; Marrinan et al., 2014). One study in 16 patients with PD and 15 healthy controls demonstrated that delayed gastric emptying in patients with PD does not appear to be due to any functional deficits of the interstitial cells of Cajal, the pacemaker cells that cause contractions of the gastric smooth muscle (Heimrich et al., 2019; Soliman et al., 2021).

While not a cause of gastroparesis *per se*, several classes of medication, including anticholinergics and some dopaminergic PD medications, may contribute to a delay in gastric emptying (Bestetti et al., 2017). The direct positive and negative effects of dopaminergic PD medications on GI function have been evaluated in a range of animal and clinical studies. Different dopaminergic medications are prescribed in PD specifically to improve motor function and reduce OFF time and are therefore hypothesized to improve GI motility (Pfeiffer et al., 2020). On the other hand, dopaminergic medications have been shown to alter the gut microbiota, associated with the development of GI symptoms, resulting in slowed GI transit (Hill-Burns et al., 2017).

A study using liquid meal scintigraphy in 51 patients with PD with and without motor fluctuations found impairment of gastric motility in 42% of subjects who had motor complications. However, they also observed that slower gastric emptying times correlated significantly with higher mean doses of dopa decarboxylase inhibitor (Bestetti et al., 2017). The authors proposed that one mechanism that may explain the delay in gastric emptying in this population was the effect of levodopa on dopaminergic receptors in the stomach and that this might be exacerbated by the use of a dopa decarboxylase inhibitor, which would increase levodopa concentrations further, resulting in an effect on drug delivery and efficacy. A study in healthy rats showed that dopamine agonist treatment resulted in a reduction in small intestinal motility and an increase in bacterial overgrowth in the distal small intestine (van Kessel et al., 2022). There was also an increase in the amount of small bowel bacteria (*Lactobacillus* species), which correlated negatively with levodopa levels in the systemic circulation, suggesting an effect on the bioavailability of levodopa.

A study comparing the prevalence and severity of GI symptoms in 163 patients with PD and 113 healthy controls using the Gastrointestinal Symptom Rating Scale (GSRS) demonstrated the important role that PD medications can play in the presentation and development of GI issues (Kenna et al., 2021). Patients with PD total daily medication dose (levodopa-equivalent daily dose; LEDD) was found to be significantly associated with an increase in all four GI symptom domain scores. More specifically, catechol-*O*-methyl transferase (COMT) inhibitors were associated with significantly decreased hypoactive GI scores, monoamine oxidase type B (MAO−B) inhibitors with significantly decreasing general GI symptoms, and amantadine with significantly decreasing the total GSRS score. In the case of MAO−B inhibitors and amantadine, it is likely that the iatrogenic GI side effects are related to an increase in dopamine bioavailability. For COMT inhibitors, the finding of an association with a reduction in hypoactive bowel symptoms is likely to be due to the fact that COMT inhibitors are known to cause diarrhea. These studies highlight that the patient’s overall medication regimen is something that will need to be considered in the management plan for patients with PD who are identified as having gastroparesis (Marrinan et al., 2014; Soliman et al., 2021).

### Impact of gastroparesis on patients with Parkinson’s disease

The development of motor complications –motor fluctuations and dyskinesias – is a recognized aspect of PD progression (Leta et al., 2019). Motor fluctuations have different subtypes, including ON–OFF phenomena (variations between periods of good motor control and poor motor control), wearing off (the effect of a dose of medication wears off before the next dose is taken), delayed time-to-ON (delayed clinical effect of the dose of medication), suboptimal ON or dose failure (no effect of the dose of medication) and result in increasing periods of daily OFF time for the patient (Jankovic, 2005). While end-of-dose wearing off is probably the most well recognized type of motor fluctuation, prolonged time to ON has been shown to be twice as long as wearing off and, therefore, a major contributor to daily OFF time in patients with PD (Merims et al., 2003). Delayed ON of the first daily dose of levodopa is known as morning akinesia, and this can significantly affect the patient’s quality of life and impair daily activities (Isaacson and Chaudhuri, 2013). Levodopa-induced dyskinesia is commonly seen in patients with PD usually after long-term levodopa treatment and can be classified as either peak-dose dyskinesia, wearing-off or OFF-period dyskinesia, or diphasic dyskinesia (Bhidayasiri and Truong, 2008; Pandey and Srivanitchapoom, 2017).

Gastroparesis can be a contributing factor in delayed time to ON in patients with PD. Since levodopa is absorbed from the small intestine, delayed emptying of the stomach has important implications for drug delivery to its absorption site. Gastric emptying time is known to be a determining factor for onset of relief of motor symptoms in PD (Nyholm and Lennernas, 2008), so if it is delayed, oral levodopa absorption can be erratic, resulting in motor fluctuations (Muller et al., 2006; Stillhart et al., 2020). This can manifest in patients with PD as delayed time-to-ON, suboptimal ON or even dose failure (Merims et al., 2003; Stocchi et al., 2019). Retention of food within the stomach can be another contributing factor to delayed ON time leading to the interaction between dietary protein and levodopa, further compromising drug absorption. It is, therefore, essential that any occurrence of gastroparesis is identified promptly and managed effectively to ensure PD motor control is not compromised.

### Recognition and assessment

The initial assessment of gastroparesis should be done by taking a careful clinical history of prevalent GI symptoms, noting those that occur most commonly, with the aim of ruling out any other conditions with similar or overlapping symptoms (Parkman et al., 2004). A study of 146 subjects with gastroparesis found that the predominant symptoms were nausea (92%), vomiting (84%), bloating (75%), early satiety (60%), abdominal pain (46%), and a similar profile has been reported in other studies (Tang and Friedenberg, 2011). Further diagnostic information can be gained by using a recognized and validated patient-reported questionnaire, the Gastric Cardinal Symptom Index (GCSI) (Table 2) (Revicki et al., 2012; Soliman et al., 2021). This asks patients to evaluate the severity of nine GI symptoms over the previous 2 weeks, scored from 0 to 5 (nausea, retching, vomiting, stomach fullness, early satiety, postprandial fullness, loss of appetite, bloating, and stomach distension). The resulting global score can assist the clinician in deciding if further interventional or imaging tests are needed. As for dysphagia, the NMSQuest may be helpful in determining the presence of gastroparesis (Chaudhuri et al., 2006). If symptoms are chronic and refractory, an upper GI endoscopy can be considered to rule out any mechanical obstruction.

Several methods are available to confirm a diagnosis of gastroparesis, but in real-world clinical practice, they may be expensive or unavailable. Breath tests offer the cheapest and least invasive option. Possible procedures include:

• Gastric emptying scintigraphy – the ‘gold standard’ gastric motility test. The patient is given a solid radiolabeled meal, and gastroparesis is confirmed if retention is >60% at 2 h and/or >10% at 4 h (Tang and Friedenberg, 2011; Soliman et al., 2021).
• ^13^C gastric emptying breath test – an indirect measure of gastric motility. The patient is given a solid meal incorporating the ^13^C isotope and the amount excreted in breath samples is measured at intervals (Tang and Friedenberg, 2011; Soliman et al., 2021).
• Wireless motility capsule – a single-use, orally-ingested, non-digestible, data recording device that can be used to measure transit time in the stomach, the small intestine, and the colon (Su et al., 2017b).

### Developing a management plan

Once a diagnosis of gastroparesis is confirmed, an appropriate management plan can be considered for the patient. Management of gastroparesis is focused on improving gastric emptying itself and methods to circumvent the inconsistency in drug absorption that may result from gastroparesis. A range of gastroparesis treatment guidelines are available to help guide therapeutic choices, although these have not been developed specifically for gastric motility issues arising in patients with PD (Parkman et al., 2004; Parrish et al., 2007; Camilleri et al., 2013). As a result, implementing clinical guidelines for gastroparesis in patients with PD is not that straightforward, as certain recommendations are not applicable in PD, for example the use of metoclopramide.

The management of gastroparesis in patients with PD, just as in the case of dysphagia, will ideally need a multidisciplinary approach with input from both the neurology/movement disorders team and gastroenterology colleagues (Barboza et al., 2015). Management options for gastroparesis in patients with PD generally fall into three categories: dietary modifications, medical management, and physical/mechanical interventions.

#### Dietary modifications

A ‘gastroparetic diet’ is a simple strategy that patients with PD can employ to help reduce their gastroparesis symptoms while maintaining adequate fluids and nutrition (Legge et al., 2020). It is suggested that patients with gastroparesis should be encouraged to eat smaller and more frequent meals spread throughout the day. They should try to avoid meals that are high in fat or indigestible fiber and eat more liquid-based meals since these seem to be less affected by gastroparesis issues. It is known that various foods, drinks and dietary supplements can impact the pharmacokinetics and pharmacodynamics of PD medications (van Kessel and El Aidy, 2019; Agnieszka et al., 2022), and this is something that is likely to be exacerbated in patients with gastroparesis where food is retained in the stomach. In the case of oral levodopa, to optimize its efficacy, the literature suggests that there are potential benefits to eating a low-fat and protein redistribution diet (concentrating the protein intake at a certain time of the day) (Agnieszka et al., 2022).

A survey of gastroparesis sufferers undertaken to identify and characterize foods-provoking or -alleviating symptoms found that foods-provoking symptoms were generally fatty, acidic, spicy, and roughage-based (Wytiaz et al., 2015). They included: orange juice, fried chicken, cabbage, oranges, sausage, pizza, peppers, onions, tomato juice, lettuce, coffee, salsa, broccoli, bacon, and roast beef. In contrast, foods shown to be tolerable were generally bland, sweet, salty, and starchy. Therefore, patients with PD with gastroparesis should choose low-fiber, soft fruits, and vegetables, ensuring they are well cooked, mashed, or liquidized (e.g., bananas, fruit purees, and vegetable juices), white carbohydrates (rice and bread), and low-fat protein sources (poultry, lean meats, and low-fat dairy products).

#### Physical activity

Regular physical activity has been shown to have substantial overall benefits for patients with PD (Ellis et al., 2021). However, studies in healthy subjects have shown that exercise can also be an important management tool for gastroparesis (Matsuzaki et al., 2016), so this is something that should be considered in the overall management of patients with PD who experience gastroparesis.

#### Medical management

##### Review of existing Parkinson’s disease and concomitant medications

Parkinson’s disease therapies and other concomitant medications taken for comorbid conditions can both contribute to, or be affected by, gastroparesis (Pfeiffer et al., 2020). Therefore, to ensure ongoing control of motor function in patients with PD, an important aspect when managing gastroparesis is ensuring the patient continues to receive sufficient, appropriate PD medication to manage their motor symptoms without exacerbating gastric motility issues. Several classes of drugs, for PD and for other conditions, are known to negatively impact gastroparesis, for example, anticholinergics and proton-pump inhibitors (Soliman et al., 2021). With this in mind, a review of the patient’s current PD medication regimen, as well as any other medications taken for concomitant diseases, should be undertaken prior to considering any specific GI medications that may be needed.

In patients with PD who have gastroparesis, efforts should be made to ensure that the clinical effect of oral dopaminergic therapy is optimized using techniques such as taking soluble or slow-release levodopa formulations, fractionating doses, or taking levodopa in combination with ascorbic acid to improve its absorption (Nagayama et al., 2004). Additionally, patients should be advised to take oral dopaminergic therapy on an empty stomach, if possible, and consider protein redistribution, where patients are recommended to take the daily protein allowance at the last meal. If these strategies prove ineffective, an alternative approach to the problem of gastroparesis in patients with PD is to avoid taking medication by the GI route and transition to non-oral therapies (Chaudhuri et al., 2016; Pfeiffer et al., 2020). These include device-aided therapies such as DBS, subcutaneous apomorphine injection, subcutaneous apomorphine infusion, and intrajejunal levodopa infusion, with other formulations in development (Leta et al., 2021). In addition, transdermal rotigotine patch has shown efficacy in reducing gastrointestinal symptoms in patients with PD who switched from oral therapy (Woitalla et al., 2015).

##### Pharmacological options to manage gastroparesis

Strategies to manage gastroparesis focus on the acceleration of gastric emptying but also on treating the various individual symptoms of gastroparesis (Tang and Friedenberg, 2011; Barboza et al., 2015; Camilleri, 2016; Soliman et al., 2021). Not all strategies have been specifically evaluated in patients with PD. Management should be individualized, focusing on the main troublesome symptom in each patient. Table 3 summarizes pharmacological options for the management of gastroparesis in PD.

**TABLE 3:** Pharmacological options for the management of gastroparesis.

Drug	Dose	Limitations
**Peripheral D2 receptor antagonist**		
Domperidone	10 mg PO. tid	• Risk of QT prolongation and cardiac arrthythmias – Follow-up electrocardiogram is advised. • Lactation. • CYP3A4 interaction.
**Motilin receptor agonist**		
Erythromycin	150–250 mg PO. tid to qid, given 30 min before each meal. 250 mg IV q 24 h	• Not recommended for long term use. • Clinical responsiveness drops after 4 weeks of oral erythromycin. • CYP3A4 interactions, antibiotic resistance. • QTc prolongation.
Azithromycin	250 mg IV q 24 h	• Not recommended for long term use. • Clinical responsiveness drops after 4 weeks of oral erythromycin. • CYP3A4 interactions, antibiotic resistance. • QTc prolongation.
**Ghrelin agonist**		
Relamorelin	100 μg by subcutaneous injection daily in the morning.	Safety and efficacy cannot be determined due to early termination of the study in PD. Side effects include headache and increased appetite.
**Selective H2 receptor antagonist**		
Nizatidine	150 mg bid	No reported adverse effects.
**Selective 5HT-4 receptor agonist**		
Mosapride	15 mg OD and increase 10–15 mg/week to 45 mg OD	No or minimal adverse effects on cardiovascular and central nervous system, because of their high binding selectivity. Side effects include abdominal pain and headache.
Prucalopride	1–2 mg OD	No or minimal adverse effects on cardiovascular and central nervous system, because of their high binding selectivity. Side effects include abdominal pain and headache.
**Cholinesterase inhibitor**		
Bethanechol/Pyridostigmine	60 mg PO. tid	Safety and efficacy cannot be determined due to no study in PD. Side effects include diarrhea, abdominal cramping, flushing, and hypersalivation.

A first-line option to accelerate gastric emptying is the peripheral dopaminergic D2 receptor antagonist, domperidone, which has a prokinetic effect and does not cross the blood–brain barrier. In contrast, metoclopramide, another dopamine D2 receptor antagonist, is able to cross the blood-brain barrier resulting in central dopamine antagonism and worsening PD, so it is, therefore, contraindicated in this setting. In clinical practice, domperidone is frequently used in patients with gastroparesis, accounting for 28% of all prescribing indications (Ehrenpreis et al., 2017). Despite its frequent use, concerns arise about its safety, with several warnings issued by many regulatory agencies (i.e., Health Canada, European Medicines Agency, Medicines and Healthcare Products Regulatory Agency in the United Kingdom) against the use of domperidone in daily doses of more than 30 mg in patients over 60 years due to potential risks of sudden cardiac death and ventricular arrhythmias (Bozzo et al., 2012; Benstetter and Harvey, 2014a; Medicines, Healthcare Products, and Regulatory Agency, 2014). The findings may be caused by domperidone’s propensity to prolong QT-interval and cardiac repolarization, which can trigger severe forms of ventricular arrhythmia (torsades de pointes) that can result in sudden cardiac death (Yap and Camm, 2003). The risk of arrhythmias may even be higher in those patients who are prescribed domperidone along with QT-interacting drugs, such as quetiapine, nortriptyline, and escitalopram, which are also frequently used in PD patients (Ehrenpreis et al., 2017). Indeed, the most recent systematic review and meta-analysis of observational studies that employed stringent quality assessment criteria has reported an increased risk of sudden cardiac death and ventricular arrhythmia with domperidone compared to non-users (Ou et al., 2021). The risk was especially evident with higher doses and in elderly individuals, as frequently observed in PD patients who nevertheless were prescribed domperidone as their first-choice anti-emetic and prokinetic agent (Lertxundi et al., 2013). A subsequent study in a specific PD population has identified a significant risk in those with a history of cardiovascular disease, but not in those without (Renoux et al., 2016). Based on this documented risk, the benefit-risk balance of domperidone should be individualized and be used at the lowest effective dose for the shortest possible duration, with the maximum treatment duration not exceeding 1 week and the maximum dosage not exceeding 30 mg daily (Benstetter and Harvey, 2014b). Notably, the use of domperidone is contraindicated in patients with severe hepatic impairment, conditions where cardiac conduction is, or could be, impaired or where there is underlying cardiac disease, and when co-administered with QT-prolonging medications or potent CYP3A4 inhibitors.

Other compounds are also known to have prokinetic effects (Ramprasad et al., 2018; Camilleri and Atieh, 2021). Erythromycin and azithromycin increase gastric antral contractions and enhance gastric emptying. In contrast to other proton-pump inhibitors, nizatidine is a H2 blocker that inhibits gastric acid secretion and acetylcholinesterase and shortens gastric emptying time. Ghrelin is a neuropeptide that binds to GH receptors, exerting prokinetic effects by accelerating gastric emptying and also improving appetite. Ghrelin agonists have therefore been evaluated as prokinetic agents in gastroparesis (Shin and Wo, 2015). Newer 5HT4 agonists could be potential prokinetic options, including mosapride and prucalopride. Other options include bethanechol, a cholinesterase inhibitor with cholinergic properties and pyridostigmine, a muscarinic agonist. However, both can cause hypotension and hypersalivation. Well-designed clinical trials of these prokinetic agents remain lacking in PD.

More recently, DA-9701, a traditional herbal medication containing two plant extracts, *Pharbitidis semen* and *Corydalis tuber* has been shown to have equivalent clinical efficacy to the established prokinetic, itopride, for the treatment of functional dyspepsia in a randomized, controlled Phase III trial (Choi et al., 2015). In PD, DA-9701 has been shown to improve gastric motility and GI symptom-related quality of life, without aggravating PD symptoms in two randomized controlled trials (Shin C. M. et al., 2018; Choi et al., 2020). Meanwhile, camicinal, a motilin receptor agonist that enhances gastric emptying, has been shown to improve levodopa bioavailability in a randomized placebo-controlled trial, leading to significant reduction in OFF time in patients with PD (Marrinan et al., 2018). If direct prokinetic treatment fails, other medications can be used to tackle the primary symptoms of gastroparesis, namely nausea and vomiting (Soliman et al., 2021). Nausea can be treated aggressively with options such as domperidone or ondansetron.

Research is ongoing into other potential pharmaceutical therapies that could improve gastric motility in patients with gastroparesis. These include gamisoyo-san decoction, a traditional Chinese medicine, that has been used to treat various GI symptoms. A study in mice with experimentally-induced GI motility dysfunction found that gamisoyo-san decoction was able to modulate bowel activity suggesting it might have potential as a gastroprokinetic agent for the treatment of GI motility issues (Shin S. J. et al., 2018).

#### Physical/mechanical interventions

In cases where medical therapies are not proving effective, physical/mechanical techniques may be an option, but most have not been specifically evaluated in PD or have shown limited efficacy in this setting. These include therapies that target the pyloric sphincter, namely Botulinum toxin injections, endoscopic pyloric dilation, and gastric endoscopic pyloromyotomy.

## Conclusion

Our article highlights that eating difficulties and other upper GI issues in PD extended beyond the most well-known symptom, constipation (Clarke et al., 2018; Kyritsis et al., 2021). Dysphagia and gastroparesis are often overlooked as manifestations of PD, but both have a significant impact on patients’ quality of life and the efficacy of PD therapy, so timely and effective management is needed.

As GI dysfunction in PD spans the discipline of neurology and gastroenterology, management of these issues should ideally comprise a collaborative multidisciplinary team approach to ensure the best outcomes for patients. However, in real-world clinical practice, such resources may not be immediately available, and therefore, it is important that neurologists managing patients with PD are aware and informed about all possible GI issues in PD, so they can confidently assess patients and initiate the management process. As this article highlights, there are simple techniques available that the neurologist or PD nurse specialist can use to assess patients with dysphagia or gastroparesis to aid in timely referral to relevant specialists.

When developing a management plan, it is essential that non-pharmacological approaches are considered very early and alongside any pharmacological interventions. The guidance summarized in this article should help neurologists with real-life planning for patients with PD experiencing dysphagia or gastroparesis and their carers. An important aspect of this is patient and carer education about GI issues in PD and what symptoms to look out for. An educational video on different GI symptoms in PD, including dysphagia and gastroparesis, is available in Supplementary Video 1 of this article.

## Author contributions

RB and WP contributed to conception and design of the study and organized the database. RB wrote the first draft of the manuscript. WP, AT, VL, SP, KC, and PP critiqued the manuscript. All authors contributed to manuscript revision, read, and approved the submitted version.

## Supplementary material

Supplementary Video 1 Educational video for Parkinson’s patients on different GI symptoms in PD, including dysphagia and gastroparesis.
